# An Aminobutyric Acid Transaminase in *Zea mays* Interacts With *Rhizoctonia solani* Cellulase to Participate in Disease Resistance

**DOI:** 10.3389/fpls.2022.860170

**Published:** 2022-04-05

**Authors:** Xiuna Guo, Jinyin Chen, Mengyi Gao, Duochuan Li

**Affiliations:** Department of Plant Pathology, Shandong Agricultural University, Taian, China

**Keywords:** *Rhizoctonia solani* AG1-IA, EG1, gamma aminobutyric acid transaminase, allergic necrosis, disease resistance

## Abstract

Corn sheath blight, caused by AG1-IA, a fusion group of *Rhizoctonia solani*, which acts as a kind of necrotrophic fungal pathogen, poses a global threat to the production of *Zea mays*. Although cellulase plays a crucial role in *R. solani* infections, how plants respond to it is still poorly understood. In this study, we identified a gamma-aminobutyric acid transaminase (GABA-T), ZmGABA-T, in *Z. mays* that interacts with a cell wall–degrading enzyme (CWDE), EG1, in the cell membrane, using yeast two-hybrid assay, co-immunoprecipitation (Co-IP), and bimolecular fluorescence complementation assays. We found that the combination of EG1 and ZmGABA-T suppressed the allergic necrosis induced by EG1. We also found that the substrate of GABA-T–GABA, can inhibit the transcription of EG1. Transient expression of ZmGABA-T inhibited *R. solani* infection in *Nicotiana benthamiana*. The homolog in *Oryza sativa*, OsGABA-T, could also interact with EG1 to suppress the allergic necrosis induced by EG1. The OsGABA-T knocked out plants displayed enhanced susceptibility to *R. solani* and showed larger lesions. In conclusion, our results suggest that ZmGABA-T inhibits allergic necrosis induced by EG1 based on the combination with EG1, producing resistance to *R. solani* infection.

## Introduction

Plants are constantly invaded by pathogens during their development. As a result, both have evolved a complex immune interaction network to fight each other called the “Invasion Model” (Kanyuka and Rudd, 2019). Pathogens are regarded as invasive molecules (IMs), and host receptors are called invasion pattern receptors. IMs are recognized by invasion pattern receptors and respond with apoplast-initiated immune responses and cytosol-initiated immune responses. Pathogen-associated molecular patterns (PAMPs) are typical IMs that induce PAMP-triggered immunity (PTI) *via* pattern recognition receptors (PRRs), leading to the accumulation of reactive oxygen species (ROS), calcium ion (Ca^2+^) level elevation, the activation of defense-related genes, and hypersensitive responses (HRs) (Dangl and Jones, 2001; Desaki et al., 2006; Boller and Felix, 2009; Zipfel, 2009). Several studies have reported that some CWDEs, such as xyloglucanase (Ma Z. et al., 2015; Gui et al., 2017; Snarr et al., 2017; Mauff et al., 2019; Shen et al., 2020), endopolygalacturonases (Zhang et al., 2014), and cellulase (Ma Y. et al., 2015; Guo et al., 2021) can act as PAMPs and induce PTI, in which their PAMP activities can be independent of their enzymatic activities.

HRs defend against most pathogens, such as pathogenicity in biotrophic and hemibiotrophic fungi, preventing further development in early infection stages. They comprise a powerful protective mechanism for plants (Stergiopoulos and de Wit, 2009). However, some necrotrophic pathogens, such as *Rhizoctonia solani*, have been known to kill plants. Necrotrophic fungi kill host cells and obtain nutrients from dead plant tissues. Successful necrotrophic pathogens rely on the secretion of hydrolytic enzymes to macerate and digest plants for their development (Doehlemann et al., 2017). A large number of genes coding for CWDEs and other hydrolytic enzymes present in the genomes of necrotrophic fungi support this notion (Amselem et al., 2011). However, the infection process of necrotrophic pathogens is complex. The burst of ROS, which probably has multiple functions, mediates the life cycle of necrotrophic pathogens. Although they are considered to be a kind of defense response, they also serve as a virulence factor, at least in necrotrophic interactions (Doehlemann et al., 2017). For example, HRs facilitate the infection of plants by *Botrytis cinerea* (Govrin and Levine, 2000). During *Sclerotinia sclerotiorum* infection, oxalic acid induces increased ROS levels in plants, which correlates to cell death, thereby promoting infection (Kim et al., 2008).

To resist infection by diverse pathogens, plants have evolved various defense mechanisms. Salicylic acid (SA) contributes to plants’ resistance to biotrophic and hemibiotrophic pathogens, whereas SA, jasmonate (JA), and ethylene (ET) all promote resistance to necrotrophic fungi (Tsuda et al., 2009). The Arabidopsis Botrytis Susceptible1 Interactor (BOI) aids disease resistance through the suppression of necrotrophic pathogens–induced cell death (Luo et al., 2010). Inhibitors of apoptosis proteins (IAPs) have a similar structure and function as BOI in animals (Vaux and Silke, 2005). Transgenic plants expressing baculovirus IAPs suppress cell death and are resistant to necrotrophic fungi (Dickman et al., 2001). A transcriptional repressor of gibberellin (GA) signaling, DELLA protein, promotes resistance to necrotrophic pathogens by altering the relative strength of SA and JA signaling (Navarro et al., 2008). The Arabidopsis pentatricopeptide repeat protein, peptidoglycan (PGN), regulates the ROS dynamic equilibrium in mitochondria to resist *B. cinerea* infection (Laluk et al., 2011). In contrast, the *Arabidopsis* BOTRYTIS-SUSCEPTIBLE1 (BOS) mutant is more susceptible to necrotrophic fungi (Mengiste et al., 2003).

*Rhizoctonia solani* acts as a necrotrophic fungus and harms gramineous crops, posing a severe threat to production. Necrotrophic fungi kill host cells and take nutrients from dead plant tissues to complete their life cycle. During this cycle, they secrete various cell necrosis–causing CWDEs and toxins to achieve a successful infection (Oliver and Solomon, 2010). There are many pathogenic factors in *R. solani*, such as the effector AGLIP1, which triggers cell death in plants and promotes disease development by inhibiting PTI, such as protection-related (PR) gene expression in *Arabidopsis thaliana* (Li et al., 2019). The RsRlpA effector is a protease inhibitor promoting the virulence of *R. solani* through suppression of the ROS burst and HR (Charova et al., 2020). The novel effector RsIA_NP8 in *R. solani* AG1-IA induces cell death and triggers defense responses in non-host plants (Wei et al., 2020). However, how plants interact with *R. solani* during the pathogenic infection process is not yet known. In this study, we identified a transaminase ZmGABA-T that interacts with EG1 and suppresses the allergic necrosis induced by it. We also found that the ZmGABA-T is located in the plasma membrane. GABA, the substrate of GABA-T, suppresses the transcription of EG1. Hence, we inferred that the GABA-T participates in the plant disease resistance process.

## Materials and Methods

### Strains and Plasmids

*Escherichia coli* T1 was used for cloning and nucleotide sequencing. *Pichia pastoris* GS115 and plasmid vector pPIC9K were used for stable expression. *Agrobacterium* GV3101, plasmid vectors pGR106 and pROKII were used for transient expression.

### Plant Material and Growth Conditions

Maize (*Zea mays*) plants (Non-gda 108 from Shandong, China, the most widely cultured hybrid in China) and *N. benthamiana* were grown at 25°C with 16 h of light and 8 h of darkness with a relative humidity of 60–70%. *Oryza sativa* (ZH11) and knockout mutants were grown at 28°C during the daytime and 26°C at night with 16 h of light and 8 h of darkness.

### Microbial Cultures

Strains of *R. solani* were cultured in PDA medium (200 g potato infusion, 20 g dextrose, and 20 g agar/L). Different GABA concentrations (0, 30, 40, 50, 60, and 70 mM) were added to the PDA medium to explore the effect of concentrated GABA on the transcription of *EG1.*

### *Agrobacterium tumefaciens*–Mediated Transient Expression on *Nicotiana benthamiana*

*Agrobacterium tumefaciens*-mediated transient expression was performed. Plasmid constructs were introduced into the *A. tumefaciens* strain GV3101. After culturing for 18 h in a liquid Luria Bertani medium at 28°C, the bacterial cells were harvested by centrifugation and resuspended in an infiltration medium (10 mM 2-morpholinoethanesulfonic acid, 10 mM MgCl_2_, 150 μM acetosyringone, pH 5.6). The optical densities (ODs) of the cell suspensions in the infiltration medium were adjusted to OD_600_ = 0.6 (the final concentration for each strain in a mixture). Thereafter, the cells were incubated for 3 h at room temperature before infiltration into leaves of 4-week-old *N. benthamiana* plants or *Nicotiana tabacum*. The leaves were harvested for protein 48 h after infiltration.

### *Agrobacterium tumefaciens*–Mediated Transient Expression on Onions

*Agrobacterium tumefaciens* strain GV3101 was cultivated with 200 μM of acetosyringone for 12 h, and the bacterial cells were harvested by centrifugation and resuspended in a Murashige and Skoog (MS) liquid medium (with 200 μM acetosyringone). The ODs of the cell suspensions in the MS medium (with 200 μM acetosyringone) were adjusted to OD_600_ = 0.6 (the final concentration for each strain in a mixture). The inner epidermis of the onion was torn off and cultured in the MS medium (with 200 μM acetosyringone) for 24 h in the dark. We placed the onion’s pre-cultured inner epidermis in the MS liquid medium (with 200 μM acetosyringone) to resuspend the bacteria solution, soaked it for 30 min, picked up a corner of the epidermis, drained the bacteria solution slightly, and reapplied it to the MS medium (with 200 μM acetosyringone), with a photoperiod of 16–18 h, and a total of 48 h at 25°C.

After the co-cultivation, small pieces of the onion’s inner epidermis were taken out and washed with sterile water to remove the attached Agrobacterium. The epidermis was spread on a glass slide, observed, and photographed under a scanning microscope (LSM800, Zeiss). The GFP was detected with excitation at 488 nm and emission at 525 nm, and mCherry was detected with excitation at 552 nm and emission at 600 nm.

### Reactive Oxygen Species Detection

3,3-Diaminobenzidine (DAB, 5 mg/mL) was used to detect ROS accumulation in *N. benthamiana*. Three days after the infection, leaves immersed in DAB were vacuumed for 30 min and then lighted for 4 h. After that, the liquid was discarded and the leaves were washed with 95% ethanol and boiled in water at 95°C. The system was allowed to stand until all the chlorophyll had dissolved, then the leaves were transferred to 50% ethanol for preservation and photographed.

### Protein Extraction and Detection

For enzyme activity determination, proteins expressed by *N. benthamiana* were extracted by phosphate buffer (1 × protein inhibitor cocktail, CWBIO). And for general purposes, proteins were extracted by lysis buffer (NP-40, Beyotime).

### Co-immunoprecipitation Assay

Leaves of *N. benthamiana* were co-infiltrated with *A. tumefaciens* carrying ZmGABA-T-HA and EG1-eGFP, OsGABA-T-HA and EG1-eGFP, NtGABA-T-HA and EG1-eGFP, and AtGABA-T-HA and EG1-eGFP. The infiltrated leaves were harvested after 2 days and then ground to powder in liquid nitrogen, and suspended in a lysis buffer (NP-40, Beyotime). Anti-GFP agarose bead suspension (AlpaLife by KanTi) was added to the protein supernatants. The mixture was incubated at 4°C for 8 h with constant end-over-end rotation. Then the beads were rinsed with washing buffer (10 mM Tris-HCl, pH 7.5, 0.5 mM EDTA, 150 mM NaCl), and the anti-GFP agarose mixtures were collected for Western blotting following SDS–PAGE by 2 × SDS loading buffer. Fusion proteins were detected by anti-HA and anti-GFP antibodies, respectively, according to the EasySee Western Blot Kit (Transgene, China) with the Chenpchemi series chemiluminescence/multicolor fluorescence/visible light gel imaging system.

### Pull-Down Assay

Recombinant EG1 was purified from *P. pastoris* GS115, AtGABA-T-eGFP and NtGABA-T-eGFP were expressed by transient expression in *N. benthamiana*, and then extracted proteins were collected on anti-GFP beads (AlpaLife by KanTi). The EG1 proteins were mixed with the beads carrying AtGABA-T or NtGABA-T at a final concentration of 10 μM in a buffer (10 mM Tris-HCl, pH 7.5, 0.5 mM EDTA, 150 mM NaCl), and incubated for 1 h at 4°C. Western blotting was then performed as described above.

### BiFC Assay and Microscopy

Contrast vectors EG1-cYFP and ZmGABA-T-nYFP were transformed into *A. tumefaciens*. Thereafter, *A. tumefaciens* was infiltrated onto *N. benthamiana* leaves. The leaves were observed under a confocal laser scanning microscope (LSM880, Zeiss) 48 h after the infiltration, and YFP was detected with excitation at 488 nm and emission at 515 nm.

Furthermore, pGR106 carried with EG1-eGFP and mCherry–ZmGABA-T were transformed into *A. tumefaciens*. Thereafter, *A. tumefaciens* were infiltrated onto *N. benthamiana* leaves. After 48 h infiltration. The leaves were observed under a confocal laser scanning microscope (LSM800, Zeiss) 48 h after the infiltration, and mCherry was detected with excitation at 552 nm and emission at 600 nm.

### Enzyme Activity Determination

Enzyme activity was measured using the 3,5-dinitrosalicylic acid (DNS) method (Sumner, 1925; Miller, 1959). To explore the influence of ZmGABA-T on EG1 enzyme activity, EG1 expressed by *P. pastoris* GS115 was co-incubated with ZmGABA-T and pGR106 expressed by *N. benthamiana* at 50°C for 30 min. Next, 1% carboxymethylcellulose sodium (CMC–Na) was used as the enzyme substrate, and phosphate buffer (pH 5.0) was used as the buffer system. Then DNS was used to determine glucose content and measure absorbance at OD_540_.

To understand the effect of GABA concentration on EG1 enzyme activity, GABA concentrations ranging from 30 to 70 mM were added into an enzymatic reaction system. EG1 expressed by *P. pastoris* GS115 was co-incubated with 1% CMC–Na and GABA at 50°C in a phosphate buffer (pH 5.0) for 30 min. Then DNS was used to determine glucose content and absorbance was measured at OD_540_.

### Quantitative PCR

*Zea mays* leaves injected with purified EG1 expressed by *P. pastoris* GS115 were harvested at different time points and ground in liquid nitrogen for RNA extraction with TRIzol. The first strand of cDNA was synthesized from 1 μg total RNA using TransScript All-in-One First Strand cDNA Synthesis SuperMix (Transgen, China). qRT-PCR was assayed by TransStart Top Green qPCR SuperMix (Transgen, China). Each PCR tube contained 10 μL of Top Green qPCR SuperMix, 25 ng of cDNA, and 0.2 μM of each primer. The thermal cycling conditions were 30 s at 94°C, followed by 40 cycles of 5 s at 94°C and 15 s at 55°C by Roche LightCycler 96.

*Rhizoctonia solani* was cultured in PDA with different GABA concentrations for 3 days and was collected in liquid nitrogen for RNA extraction and qRT-PCR as described above.

For biomass measurement, total DNA was extracted by the Plant Genomic DNA Kit (TIANGEN, China), and qPCR was assayed by the TransStart Top Green qPCR SuperMix (Transgen, China).

### Rice Transformation

CRISPR/Cas9-mediated gene-editing vectors were constructed according to previous reports (Ma et al., 2016). Transgenic experiments were performed using *A. tumefaciens–mediated* callus transformation in the *japonica* variety ZH11.

## Results

### EG1 Interacts With ZmGABA-T

**FIGURE 1:**
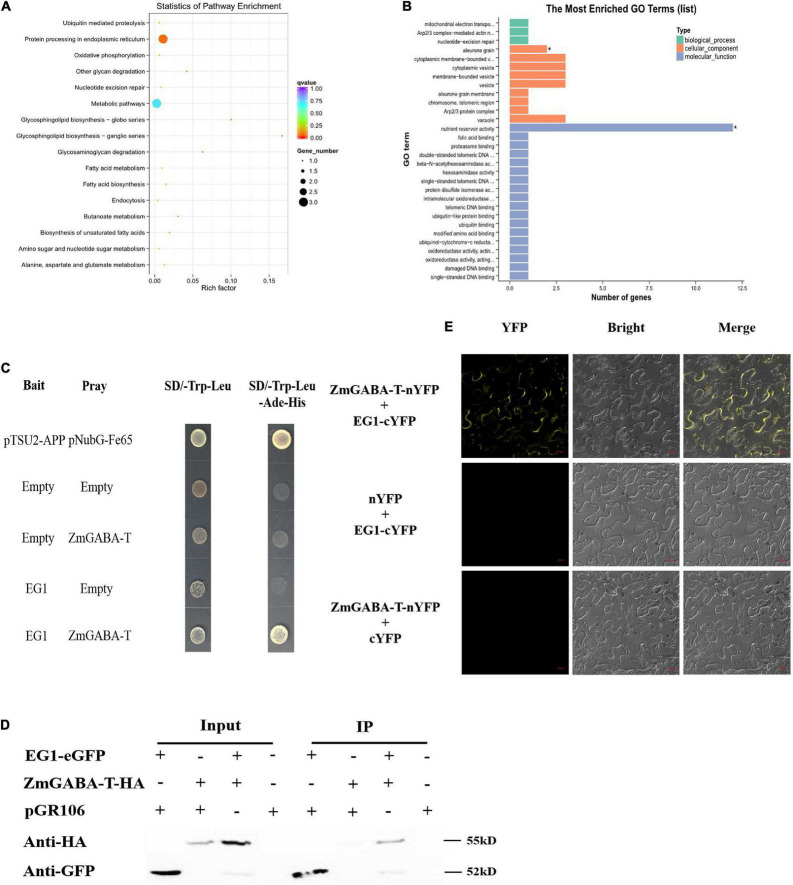
EG1 interacts with ZmGABA-T. **(A)** Biological function pathways of candidate genes. There are 43 genes in total. And all the 43 genes were classified into 16 types according to their functions: Ubiquitin-mediated proteolysis, protein processing in the endoplasmic reticulum, oxidative phosphorylation, other glycan degradation, nucleotide excision repair, metabolic pathways, glycosphingolipid biosynthesis–globo series, ganglio series, glycosaminoglycan degradation, fatty acid metabolism, fatty acid biosynthesis, endocytosis, butanoate metabolism, biosynthesis of unsaturated fatty acids, amino sugar, and nucleotide sugar metabolism, and alanine, aspartate, and glutamate metabolism. **(B)** Gene Ontology (GO) analysis of candidate genes. The function of genes is divided into three parts: Biological process, cellular component, and molecular function. And the genes function of nutrient reservoir activity has the largest number. Enriched P-values less than 0.05 are marked with “*”. **(C)** Interactions between EG1 with ZmGABA-T in the DUAL membrane yeast 2 hybrid system. Yeast NMY32 cells cotransformed with bait and prey vectors were grown on QDO (SD/–Ade/–His/–Leu/–Trp) medium. The combination of pTSU2-APP and pNubG-Fe65 was used as a positive control, while the combination of pTSU2-APP and pPR3N was used as the negative control. **(D)** Interactions between EG1 with ZmGABA-T in *Nicotiana benthamiana*. pGR106 carried EG1-EGFP and ZmGABA-T-HA, respectively, were coexpressed in *Nicotiana benthamiana.* Around 48 h after infiltration, immunoprecipitates obtained from whole-cell extracts using anti-GFP trap beads were analyzed by immunoblotting with anti-HA and anti-GFP antibodies. This experiment was repeated three times with the same results. **(E)** BiFC assay in *Nicotiana benthamiana.* Bars = 20 μm.

To find the protein that interacts with EG1 in plants, we identified potential host protein targets using DUAL membrane yeast two-hybrid (Y2H) assays. We independently screened the EG1 forms as baits against a prey library from RNA prepared at 72 h postinoculation of *Z. mays* injected with EG1 protein. A total of 43 positive colonies were captured by the bait EG1 (Figures 1A,B).

According to the analysis of results of the 43 positive colonies, we found that the protein that was most likely to interact with EG1 was ZmGABA-T and chose to make it the focus of our study. We linked the EG1 without its signal peptide with a bait construct because the secreted protein EG1 owned the signal peptide. We also connected the ZmGABA-T to the prey construct. Point-to-point verification showed that all transformants grew on the SD/-Trp-Leu, but only yeasts containing both EG1 and ZmGABA-T could grow on the SD/-Trp-Leu-Ade-His media (Figure 1C).

To prove that EG1 can interact with ZmGABA-T in plants, co-immunoprecipitation (co-IP) assays were performed by transient expression in *N. benthamiana*. The proteins were pulled down with GFP beads and were all present in the relevant input samples except for ZmGABA-T-HA, which was co-immunoprecipitated in the presence of EG1-eGFP (Figure 1D).

Then EG1 and ZmGABA-T were fused to the construct with yellow fluorescent protein (YFP), which was divided into N-terminal and C-terminal to generate EG1-cYFP or ZmGABA-T-nYFP. In addition, the two empty constructs, nYFP and cYFP, were used as a negative control. Co-expression of EG1-cYFP and ZmGABA-T-nYFP in *N. benthamiana* resulted in YFP fluorescence signals located in the cytoplasmic membrane 72 h post-infiltration (hpi). The EG1-cYFP/nYFP and ZmGABA-T-nYFP/cYFP combinations failed to fluoresce, demonstrating that neither EG1 nor ZmGABA-T produced non-specific fluorescence in *N. benthamiana* (Figure 1E). All these results indicate that EG1 interacts with ZmGABA-T in plants.

**FIGURE 2:**
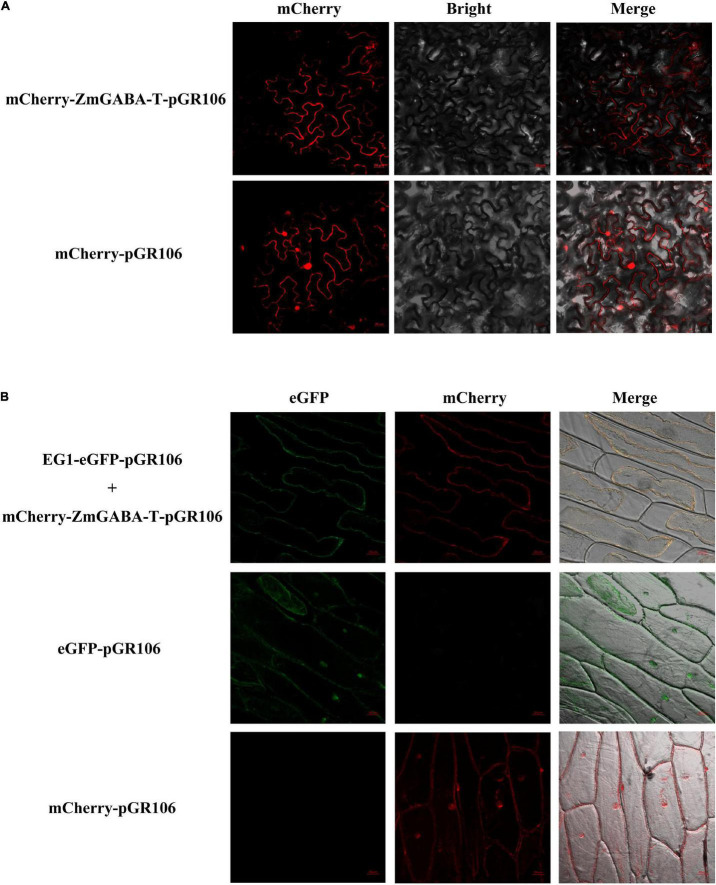
Subcellular localization of ZmGABA-T and EG1 in *Nicotiana benthamiana* and onions. **(A)** Subcellular localization of ZmGABA-T. mCherry fused with ZmGABA-T were transiently expressed by *A. tumefaciens* in *Nicotiana benthamiana* 48 h after infiltration. **(B)** Co-localization of EG1 and ZmGABA-T. For coexpressed, EG1 fused with eGFP and mCherry fused with ZmGABA-T were coexpressed in onions 48 h after infiltration. After 48 h of co-cultivation on MS medium and plasmolysis with 1 M NaCl to induce cytoplasmic separation, both red and green fluorescent signals were found on the cell membrane. The infiltrated *N. benthamiana* leaves and onion’s epidermal cells were observed *via* fluorescence microscopy. The eGFP and mCherry were used as check samples. The merged image of two fluorescence signals is shown on the right. Bars = 20 μm.

We fused mCherry to the N-terminal of ZmGABA-T, with the transient expression of mCherry–ZmGABA-T in *N. benthamiana.* This resulted in red fluorescence signals in the cytoplasmic membrane 48 hpi. The plasmolysis of the onion epidermal cells returned similar results, the EG1-eGFP colocalized with mCherry–ZmGABA-T in the onions cell membrane (Figure 2).

### ZmGABA-T and Gamma-Aminobutyric Acid Transaminase Work Together to Suppress Allergic Necrosis Induced by EG1

**FIGURE 3:**
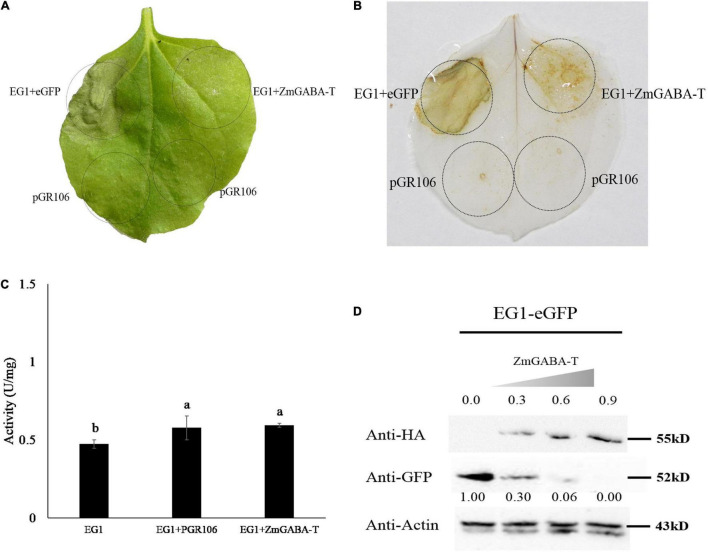
ZmGABA-T suppresses allergic necrosis induced by EG1 according to promoting the degradation of EG1, rather than inhibit the enzyme activity of EG1. **(A)** Transient expression of EG1 and ZmGABA-T in *Nicotiana benthamiana*. Transient expression was operated in *Nicotiana benthamiana* leaves injected with *Agrobacterium* carrying the indicated genes, pGR106, eGFP, EG1, and ZmGABA-T. eGFP and EG1 were coexpressed on the left of the leaf, and ZmGABA-T and EG1 were coexpressed on the right. Experiments were repeated three times at least with similar results. **(B)** ROS detection of EG1 and ZmGABA-T. The transient expression of *Nicotiana benthamiana* leaves inoculated with eGFP and EG1, ZmGABA-T and EG1 were dyed with 5 mg/mL DAB (3,3-diaminobenzidine). Transient expression was assessed in *Nicotiana benthamiana* leaves from 4-week-old plants 5 days after agroinfiltration, and pGR106 empty vector was used as a control. Experiments were repeated three times at least with similar results. **(C)** Enzyme activity determination of EG1 and ZmGABA-T. Co-incubation EG1 (1 μM) expressed by *Pichia pastoris* GS115 with pGR106 and ZmGABA-T expressed by *Nicotiana benthamiana* (2 mg/mL) separately at 50°C for 30 min, then dinitrosalicylic acid (DNS) was used to determine glucose content, and measured absorbance at OD_540_, thereby calculated enzyme activity of EG1. The results of the experiment were repeated three times at least. Lower-case letters indicate statistically significant differences (*P* < 0.05). **(D)** Co-expression of EG1-eGFP and ZmGABA-T-HA in *Nicotiana benthamiana*. The concentration of inoculation about ZmGABA-T was increased sequentially from OD_600_ = 0 to 0.9, while the concentration of EG1 was OD_600_ = 0.5. The numbers in the first row indicated the inoculation concentration of ZmGABA-T-HA. The numbers in the second row indicated the gray value of EG1. The results of the experiment were repeated three times at least.

Five days after the EG1 and ZmGABA-T were co-injected into the *N. benthamiana*, we found that the allergic necrosis caused by EG1 was weakened (Figure 3A). The ROS detected by 3,3-di-aminobenzidine (DAB) also showed weakened symptoms (Figure 3B).

To explore why the allergic necrosis was suppressed, the EG1 and ZmGABA-T were expressed by yeast and *N. benthamiana*, respectively. Then, proteins were used to detect the enzyme activity by DNS. After enzymatic reaction for 30 min at 50°C, there was no significant difference in the specific vitality of EG1 in the presence of ZmGABA-T. Therefore, we concluded that ZmGABA-T could not affect EG1 enzyme activity (Figure 3C).

Then the co-expressed proteins by *N. benthamiana* were extracted. A Western blotting analysis showed that the expression of EG1-eGFP was significantly reduced in the presence of ZmGABA-T–HA (Figure 1D). To prove the results, we increased the concentration of ZmGABA-T—-HA gradually and then co-expressed ZmGABA-T–HA and EG1-eGFP in *N. benthamiana.* The result showed that the expression of EG1-eGFP was reduced because of the increased concentration of ZmGABA-T–HA (Figure 3D). The above results proved that ZmGABA-T promotes EG1 degradation and suppresses the EG1-induced allergic necrosis in *N. benthamiana*.

**FIGURE 4:**
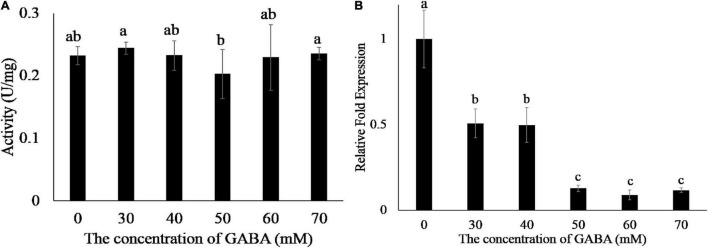
GABA does not affect the enzyme activity of EG1 but suppresses the transcription of *EG1*. **(A)** Enzyme activity determination of EG1 after adding different concentrations of GABA. Different concentrations of GABA were added to the enzymatic reaction system, and DNS was used to detect the content of glucose. The experiments were repeated three times with the same results. Lowercase letters indicate statistically insignificant differences (*P* > 0.05). **(B)** The effect of different concentrations of GABA on the transcription of *EG1*. Different concentrations of GABA were added to the Potato Dextrose Agar (PDA) medium, then *Rhizoctonia solani* was grown on PDA medium with GABA for 3 days. Total RNA was extracted 3 days after training. Then qRT-PCR was used to detect the transcription level of *EG1*. The results of the experiment were repeated three times at least. Lower-case letters indicate statistically significant differences (*P* < 0.05).

γ-Aminobutyric acid (GABA) plays an important role in the metabolism of amino acids (Ludewig et al., 2008). We added different concentrations of GABA to the EG1 enzymatic reaction system and found that GABA did not affect EG1 enzyme activity (Figure 4A). Thereafter, we added GABA to the culture medium of *R. solani*, and the transcription of *EG1* was detected by the qPCR. However, we were surprised to see that increased GABA concentration led to decreased *EG1* transcription (Figure 4B). Based on this result, we concluded that GABA repressed *EG1* transcription during *R. solani* infection. All these results prove that ZmGABA-T and GABA work together to inhibit allergic necrosis induced by EG1.

### ZmGABA-T Suppresses the Infection of *Rhizoctonia solani*

**FIGURE 5:**
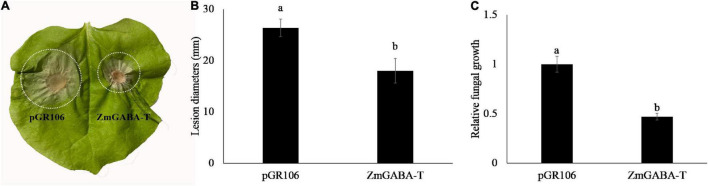
Expression of ZmGABA-T can inhibit *Rhizoctonia solani* infection. **(A)** *Rhizoctonia solani* infection on *Nicotiana benthamiana*. *N. benthamiana* leaves were inoculated by *Rhizoctonia solani* AG1-IA after transient expression of pGR106 vector and ZmGABA-T at 36 h, and photographed at 72 h post-inoculation. **(B)** Lesion diameters of infected *Nicotiana benthamiana* leaves. Lesion diameters were calculated from three independent biological replicates. Error bars represent ± SD of at least six leaves each. Lower-case letters indicate statistically significant differences (*P* < 0.05). **(C)** Quantitative PCR was performed for quantification of relative fungal growth on inoculated leaves 2^(Ct_NbActin–Ct_Rsdna)^. The values represent the means ± SD of six independent tests. Lower-case letters indicate statistically significant differences (*P* < 0.05).

To explore the function of ZmGABA-T, the leaves of *N. benthamiana*, which have transiently expressed ZmGABA-T 36 h after, were inoculated with *R. solani*. Leaves that expressed pGR106 were used as a negative control. Infection lesion sizes were recorded for comparison 4 days after inoculation with *R solani*. We found that, compared with the negative control, leaves that expressed ZmGABA-T showed smaller lesions (Figures 5A,B). Measurement of fungal DNA by quantitative PCR was used to quantify fungal colonization of the host tissues. There was a fivefold reduction in the fungal colonization of the host in leaves that had transiently expressed ZmGABA-T (Figure 5C).

### EG1 Interacts With OsGABA-T Specifically

**FIGURE 6:**
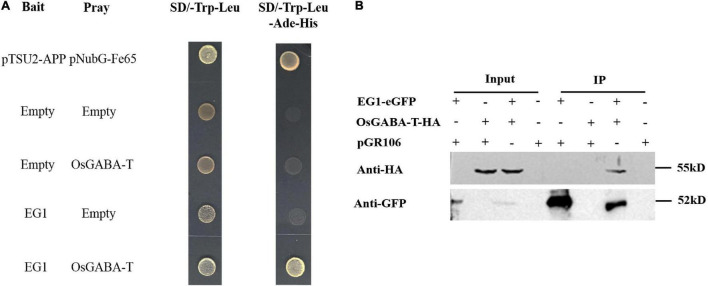
EG1 interacts with OsGABA-T. **(A)** Interactions between EG1 with OsGABA-T in the DUAL membrane yeast 2 hybrid system. Yeast NMY32 cells cotransformed with bait and prey vectors were grown on QDO (SD/–Ade/–His/–Leu/–Trp) medium. The combination of pTSU2-APP and pNubG-Fe65 was used as a positive control, while the combination of pTSU2-APP and pPR3N was used as the negative control. **(B)** Interactions between EG1 with OsGABA-T in *Nicotiana benthamiana*. pGR106 carried EG1-EGFP and OsGABA-T-HA were coexpressed in *Nicotiana benthamiana*; 48 h after infiltration, immunoprecipitates obtained from whole-cell extracts using anti-GFP trap beads were analyzed by immunoblotting with anti-HA and anti-GFP antibodies. This experiment was repeated three times with similar results.

To explore whether EG1 interacts with ZmGABA-T specifically, we compared different kinds of GABA-T around *Z. mays*, *O. sativa*, *Arabidopsis thaliana*, and *N. tabacum* (Supplementary Figure 1A), all of which had a Pyridoxal-5′-phosphate–binding domain (Ser/Thr-X-X-Lys). Yeast two-hybrid assay showed that OsGABA-T can interact with EG1. Co-IP proved that OsGABA-T interacts with EG1 weakly in *N. benthamiana* (Figure 6), and pull-down assay showed that EG1 interacts with neither AtGABA-T nor NtGABA-T (Supplementary Figures 1B,C).

**FIGURE 7:**
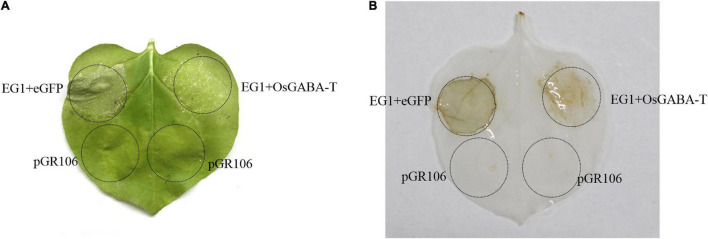
OsGABA-T suppresses allergic necrosis induced by EG1. **(A)** Transient expression of EG1 and OsGABA-T in *Nicotiana benthamiana*. Transient expression was operated in *Nicotiana benthamiana* leaves injected *Agrobacterium* carrying the indicated genes, pGR106, eGFP, EG1, and OsGABA-T. eGFP and EG1 were coexpressed on the left of the leaf, OsGABA-T and EG1 were coexpressed on the right. Experiments were repeated three times at least with similar results. **(B)** ROS detection of EG1 and OsGABA-T. The transient expression of *Nicotiana benthamiana* leaves inoculated with eGFP and EG1, and OsGABA-T and EG1 were dyed with 5 mg/mL 3,3-diaminobenzidine (DAB). Transient expression was assessed in *Nicotiana benthamiana* leaves from 4-week-old plants 5 days after agroinfiltration, and pGR106 empty vector was used as a control. Experiments were repeated three times at least with similar results.

So we continued to explore if OsGABA-T has the same function as ZmGABA-T. Transient expression of OsGABA-T and EG1 showed that the allergic necrosis induced by EG1 was attenuated (Figure 7A). The ROS detected by DAB also showed an obvious weakening of the symptoms (Figure 7B).

### Knockout of OsGABA*-T* Reduces Rice Resistance to *Rhizoctonia solani*

**FIGURE 8:**
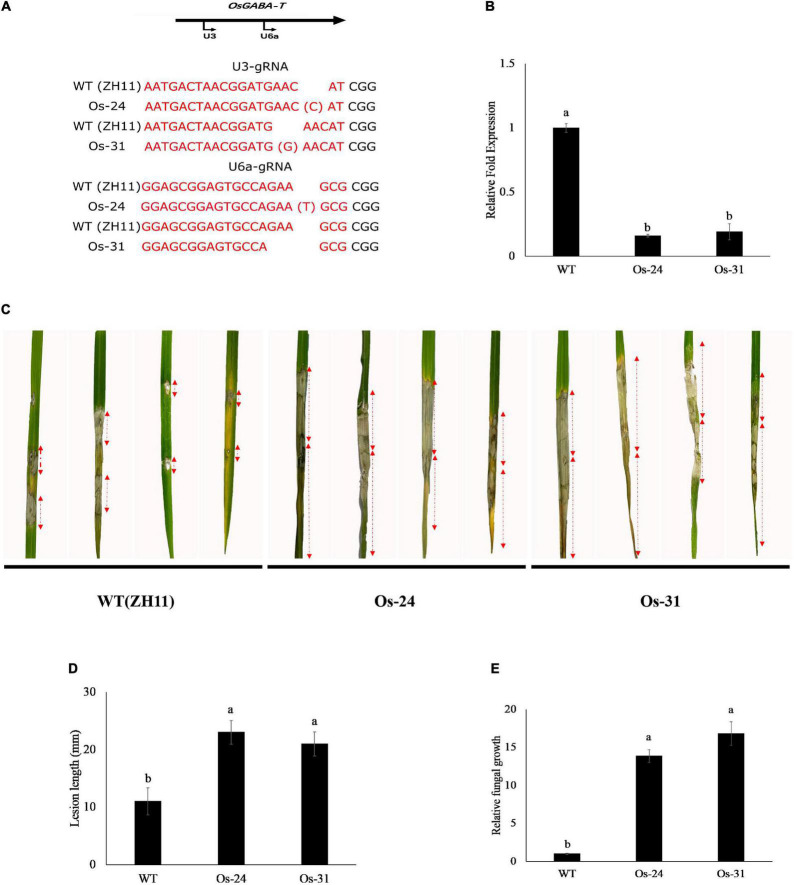
Knockout of *OsGABA-T* reduces rice disease resistance to *Rhizoctonia solani*. **(A)** Sequence of gRNA target of rice. Red color represents the target sequence. **(B)** *OsGABA-T* transcription levels in wild-type ZH11 and *OsGABA-T* knocked out rice lines, Os-24 and Os-31. Error bars represent ± SD of at least three independent tests. Lower-case letters indicate statistically significant differences (*P* < 0.05). **(C)** Inoculation of wild-type ZH11 and *OsGABA-T* knocked out lines Os-24 and Os-31 with *Rhizoctonia solani* AG1-IA. Images were taken 72 h after inoculation. **(D)** Lesion length of infected rice leaves. Lesion length was calculated from three independent biological replicates. And the red arrows indicated lesion length. Error bars represent ± SD of at least six leaves each. Lower-case letters indicate statistically significant differences (*P* < 0.05). **(E)** Quantitative PCR was performed for quantification of relative fungal growth on inoculated leaves 2^(Ct_OsUbiq–Ct_RsDNA)^. Error bars represent ± SD of 6 independent tests. Lower-case letters indicate statistically significant differences (*P* < 0.05).

Two independent transgenic rice lines whose *OsGABA-T* were knocked out by CRISPR (Os-24 and Os-31) were tested for OsGABA-T expression. A cytosine (C) or guanine (G) residue was inserted into the putative U3-gRNA target site, and thymine (T) was inserted into the putative U6a-gRNA target site or a guanine–adenine–adenine (GAA) sequence was lost from the U6a-gRNA target site (Figure 8A). Both caused an 80% more reduction in OsGABA-T expression than the non-transformed line ZH11 wild type at 4 weeks (Figure 8B). Mutant plants exhibited reduced resistance against *R. solani* (Figure 8C). The measurement of fungal DNA by qPCR was used to quantify the fungal colonization of the host tissue. Compared with the wild type, the lesion length of Os-24 and Os-31 were significantly increased (Figure 8D). There was a 15-fold increase in the fungal colonization of the host in Os-24 and Os-31 plants (Figure 8E). All the results showed that mutant plants increased sensitivity to *R. solani*.

## Discussion

The HR refers to cell death induced by pathogenic infections and is a classical indicator of resistance. Cell death has markedly different functions in plant responses to necrotrophs and biotrophs. HR is thought to confine pathogens by abolishing nutrient supply, thereby limiting the growth of pathogens in biotrophs (Mengiste, 2012). However, cell death also occurs at the early stage of necrotrophic pathogens infection and promotes further infection (Govrin et al., 2006; van Kan, 2006). Necrotrophic fungi, including *R. solani*, kill the cells and tissues of the host plant and then draw nutrients from them. Successful necrotrophs rely on a large number of hydrolytic enzymes for the maceration and utilization of the plant as their source of nutrition (Doehlemann et al., 2017). As a countermeasure, plants develop surveillance systems to perceive necrotrophic fungi and activate their innate immunity, like the signal of chitin perception, the regulation of mitogen-activated protein kinase, and the modification of histones (McGrath et al., 2005; Veronese et al., 2006; Kemmerling et al., 2007; Berr et al., 2010; Lenz et al., 2011; Li et al., 2012; Zhang et al., 2012). In our previous study, we proved that the cellulase EG1 secreted by *R. solani* can act as a PAMP that participates in plant immune responses (Ma Y. et al., 2015; Guo et al., 2021). In this study, we identified ZmGABA-T, a GABA-T from *Z. mays* through Y2H screening, and the interaction between ZmGABA-T and EG1 was verified by Y2H, CoIP, and BiFC assays (Figure 1).

GABA-T catalyzes the breakdown of GABA to succinic semialdehyde (SSA), playing a key role in its life cycle and metabolism (Cauwenberghe and Shelp, 1999; Cauwenberghe et al., 2002). In *A. thaliana*, full-length AtGABA-T contains an N-terminal targeting pre-sequence that is 36 amino acids long and is both sufficient and necessary for targeting enzymes to mitochondria (Clark et al., 2009a). In the tomato plant *Solanum lycopersicum* L., three GABA-Ts owned highly similar sequences in their coding regions, except their N-terminal regions, leading to distinct subcellular localizations in the mitochondrion, plastid, or cytosol (Clark et al., 2009b). In *O. sativa*, four putative GABA-T genes exhibit a high amino acid identity (73–82%), but they differ in length at the N-terminal region, located in different organelles (Shimajiri et al., 2013). Our study revealed that ZmGABA-T in *Z. mays* is located in the cell membrane (Figure 2A).

In *A. thaliana* and *Z. mays*, plant elicitor peptides (PEPs) play a key role in plant immunity (Yamaguchi and Huffaker, 2011; Bartels and Boller, 2015). PEPs without N-terminal secretion signals can be released to the extra-plasma by unidentified mechanisms and identified by plant elicitor peptide receptors (PEPRs) on the plasma membrane (Yamaguchi et al., 2006; Yamaguchi et al., 2010; Yamaguchi and Huffaker, 2011). ZmGABA-T also lacks transmembrane structure and does not contain a signal peptide. During the infection of *R. solani*, ZmGABA-T can interact with EG1 in the cell membrane (Figure 2B). Therefore, we concluded that an unknown protein can interact with it in cells and anchor it to the cell membrane.

Research has shown that the regulatory actions of GABA-T are closely related to immune responses to pathogens (Wu et al., 2006). Our results showed that the transient expression of GABA-T in *N. benthamiana* decreased the level of ROS triggered by EG1 and further suppressed the allergic necrosis induced by EG1 (Figures 3A,B, 7). In addition, we proved that the reduced allergic necrosis and ROS can be attributed to the promotion of EG1 degradation by ZmGABA-T rather than the inhibition of EG1 activity in *N. benthamiana* (Figures 3C,D).

GABA is a four-carbon, ubiquitous, non-proteinogenic amino acid. In animals, it is a signaling molecule that functions as an inhibitory neurotransmitter. In plants, abiotic stress (hypoxia, heat, cold, drought, mechanical wounding) or biotic stress (wounding due to herbivory and infection) results in the rapid accumulation of GABA. In addition, GABA can also act as a signaling molecule in plant defense (Bown and Shelp, 2016). Our results showed that GABA can suppress the transcription of *EG1* while not affecting its enzymatic activity (Figure 4). Thus, we conclude that GABA-T and GABA can directly or indirectly inhibit the expression of EG1, and therefore the production of ROS and allergic necrosis.

The transient expression of ZmGABA-T inhibited *R. solani* infection, and the size of lesions and the biomass of pathogenic fungi were reduced (Figure 5). When the homologous sequence of ZmGABA-T in *O. sativa* was knocked out, the resistance of knocked out plants to *R. solani* was significantly weakened (Figure 8). These results suggest that GABA-T may positively regulate disease resistance against *R. solani* in rice.

In plants, GABA-T is involved in the metabolism of GABA, which is a significant component of the free amino acid pool in most prokaryotic and eukaryotic organisms (Bown, 1997; Shelp et al., 1999). GABA is metabolized mainly *via* the GABA-shunt pathways. GABA production from glutamate is catalyzed by glutamate decarboxylase; whereas GABA-T catalyzes the conversion of GABA to SSA, which is then oxidized to succinate due to succinic semialdehyde dehydrogenase. Thus, the carbon skeleton of GABA enters the tricarboxylic acid cycle (Tuin and Shelp, 1994; Shelp et al., 2012). Other studies have shown that GABA-T (GABA shunt) may play a role in restricting the levels of allergic necrosis during host and pathogen interaction (Wu et al., 2006).

In summary, our study revealed that GABA-T in *Z. mays* can target the CWDE EG1 and act as a positive defense regulator that can suppress the accumulation of ROS and allergic necrosis caused by EG1. Future studies will focus on investigating the protein interactions with ZmGABA-T and the receptor that interacts with EG1 in the plasma membrane.

## Data Availability Statement

The datasets presented in this study can be found in online repositories. The names of the repository/repositories and accession number(s) can be found below: Sequence data from this article can be found in the GenBank/EMBL data libraries under the following accession numbers: ZmGABA-T (XM_008670668.1), OsGABA-T (AF297651.1), NtGABA-T (XM_016626518.1), and AtGABA-T (NM_113117.4).

## Author Contributions

XG and DL designed the experiments, analyzed the data, and contributed to the drafting and revision of the manuscript. XG, JC, MG, and DL performed the experiments. All authors read and approved the final manuscript.

## Conflict of Interest

The authors declare that the research was conducted in the absence of any commercial or financial relationships that could be construed as a potential conflict of interest.

## Publisher’s Note

All claims expressed in this article are solely those of the authors and do not necessarily represent those of their affiliated organizations, or those of the publisher, the editors and the reviewers. Any product that may be evaluated in this article, or claim that may be made by its manufacturer, is not guaranteed or endorsed by the publisher.
