# Supplementary material for: An Aminobutyric Acid Transaminase in Zea mays Interacts With Rhizoctonia solani Cellulase to Participate in Disease Resistance
Source: Front Plant Sci. 2022 Apr 5;13:860170. doi: 10.3389/fpls.2022.860170 (PMC9037289; doi:10.3389/fpls.2022.860170)
Supplement: Supplementary file 1 [file Table_1.docx]

**SUPPLEMENTARY MATERIAL**

**Supplementary Table S1** Primer used in this study

| **Primer** | **Sequence 5’ to 3’** | **Purpose** |
| --- | --- | --- |
| ZmGABA-T-3N-F | AAGGCCATTACGGCC ATGATGATCGCGCGA | For pPR3N-ZmGABA-T consturction |
| ZmGABA-T-3N-R | CCGGCCGAGGCGGCCCC GTTCTTCTTGGACTG |  |
| EG1-BT-F | AAGGCCATTACGGCCCAGTCCGGTACGGGCGCTACG | For pBT3SUC -EG1 construction |
| EG1-BT-R | CCGGCCGAGGCGGCCCCCTTGCGAGAGCATCCGGTGA |  |
| OsGABA-T-3N-F | AAGGCCATTACGGCC ATGGTGATTG CGCGC | For pPR3N-OsGABA-T construction |
| OsGABA-T-3N-R | CCGGCCGAGGCGGCCCC ATTGTTCTTCTTGGA |  |
| EG1-pGR106-F | GCACCAGCTAGC ATCGAT ATGAAGCTCG TCATT | For pGR106-EG1-EGFP construction |
| EG1-EGFP-R | TTGCTCAC CTTGCGAGAGCATCCGGTG |  |
| EGFP-EG1-F | TGCTCTCGCAAG GTGAGCAAGGGCGAGGAGC |  |
| EGFP-pGR106-R | AACCGTTCATCGGCG GTCGAC TTACTTGTACAGCTCGTCCATGCC |  |
| ZmGABA-T-pGR106-F | ATG ATCGAT ATGATGATCGCGCGACG | For pGR106-ZmGABA-T-HA construction |
| ZmGABA-T-HA-pGR106-R | ATAAGAAT GCGGCCGC TCA AGCGTAATCTGGAACATCGTATGGGTA GTTCTTCTTGGACTGCAGG |  |
| OsGABA-T-pGR106-F | ATG ATCGAT ATGGTGATTG CGCGCGG | For pGR106-OsGABA-T-HA construction |
| OsGABA-T-HA-pGR106-R | ATAAGAAT GCGGCCGC TCA AGCGTAATCTGGAACATCGTATGGGTA ATTGTTCTTCTTGGA |  |
| AtGABA-T-pROKII-F | ACGGGGGACTCTAGA GGATCC ATGGTCGTTATCAAC | For pROKII-AtGABA-T construction |
| AtGABA-T-pROKII-R | GCCCTTGCTCACCAT GGTACC CTTCTTGTGCTGAGC |  |
| NtGABA-T-pROKII-F | ACGGGGGACTCTAGA GGATCC ATGGCCAT GATTTC | For pROKII-AtGABA-T construction |
| NtGABA-T-pROKII-R | GCCCTTGCTCACCAT GGTACC CTTCTGAGACTT |  |
| EG1-CE-F | ACTAGTGGATCC ATCGAT ATGAAGCTCG TCATTTC | For pSPYCE-EG1 construction |
| EG1-CE-R | GGTACCCTCGAG GTCGAC CTTGCGAGAGCATC |  |
| ZmGABA-T-NE-F | ACTAGTGGATCC ATCGAT ATGATGATCG CGCGAC | For pSPYNE-ZmGABA-T construction |
| ZmGABA-T-NE-R | GGTACCCTCGAG GTCGAC GTTCTTCTTGGACTGCAGG |  |
| Mcherry-pGR106-F | TCAGCACCAGCTAGC ATCGAT ATGTTGAGCAAGGGCGAGG | For pGR106-Mcherry-ZmGABA-T construction |
| Mcherry-ZmGABA-T-R | GCGCGATCATCATCTTGTACAGCTCGTCCATGCC |  |
| ZmGABA-T-Mcherry-F | GTACAAG ATGATGATCGCGCGACGC |  |
| Mcherry-pGR106-R | AACCGTTCATCGGCG GTCGAC CTAGTTCTTCTTGGACTGCAGGG |  |
| EG1-9k-F | GAAGCTTACGTA GAATTC CAGTCCGGTA CGGGC | For pPIC9K-EG1-His construction |
| EG1-9K-His-R | CGAATTAATT CGCGGCCGC ATGGTGATGGTGATGGTG CTTGCGAGAGCATCCGGTGAT |  |
| Os-U3-F | ggcaATGACTAACGGATGAACAT | For CRISPR-Cas9-construction |
| Os-U3-R | aaacATGTTCATCCGTTAGTCAT |  |
| Os-U6a-F | gccgGAGCGGAGTGCCAGAAGCG | For CRISPR-Cas9-construction |
| Os-U6a-R | aaacCGCTTCTGGCACTCCGCTC |  |
| qOsActin-F | TCTGTATGCCAGTGGTCGT | qRT-PCR primer for *Oryza sativa* internal control gene |
| qOsActin-F | GCCGTTGTGGTGAATGAG |  |
| qOsGABA-T-F | TCAGTACCATCGCCCCAGTA | qRT-PCR primer for OsGABA-T |
| qOsGABA-T-R | CAGCTGTGAATGGTGCCAAC |  |
| qEG1-F | CAGGTGGGGATCTCGGAAAC | qRT-PCR assay for EG1 |
| qEG1-R | TAGAAGGTGCCCCGTACTGA |  |
| qRs 18s-F | ATGATAACTCGACGGATCGC | qRT-PCR primer for internal control gene Rs18s |
| qRs 18s-R | CTTGGATGTGGTAGCCGT |  |
| qNBactin-F | TTGGCTTACATTGCTCTTG | qPCR primer for internal control gene NBactin |
| qNBactin-R | TCATTGATGGTTGGAACAG |  |
| qRsDNA-F | GCCTTTTCTACCTTAATTTGGCAG | qPCR primer for *Rhizoctonia solani* internal control gene |
| qRsDNA-R | GTGTGTAAATTAAGTAGACAGCAAATG |  |
| qOsUbiq-F | GTGGCCAGTAAGTCCTCAGC | qPCR primer for *Oryza sativa* internal control gene |
| qOsUbiq-R | GAAACGGGACACGACCAAGG |  |
