# Supplementary material for: An Aminobutyric Acid Transaminase in Zea mays Interacts With Rhizoctonia solani Cellulase to Participate in Disease Resistance
Source: Front Plant Sci. 2022 Apr 5;13:860170. doi: 10.3389/fpls.2022.860170 (PMC9037289; doi:10.3389/fpls.2022.860170)
Supplement: Supplementary file 2 [file Data_Sheet_1.docx]

**SUPPLEMENTARY MATERIAL**

**Supplementary Figure S1.**

A


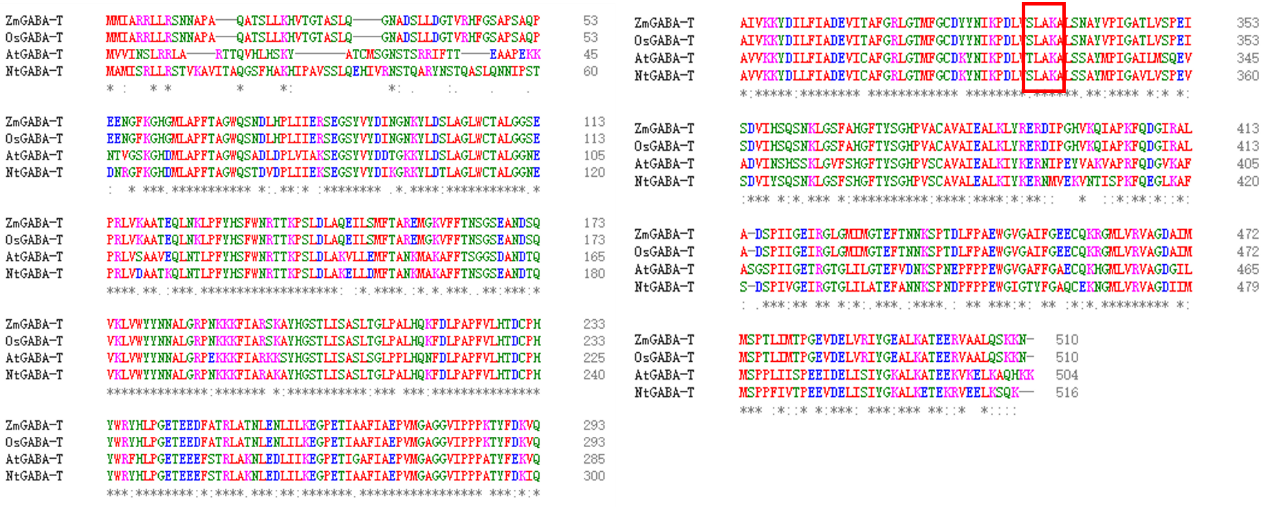


B


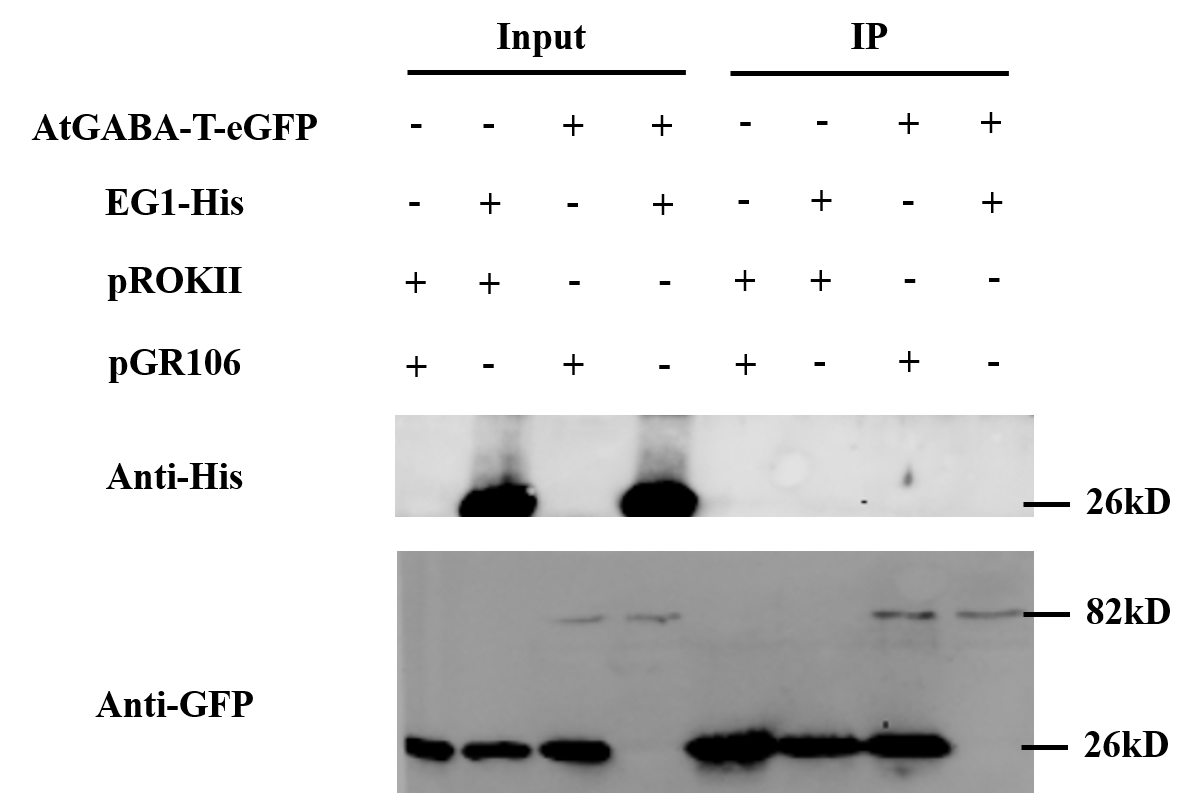


C


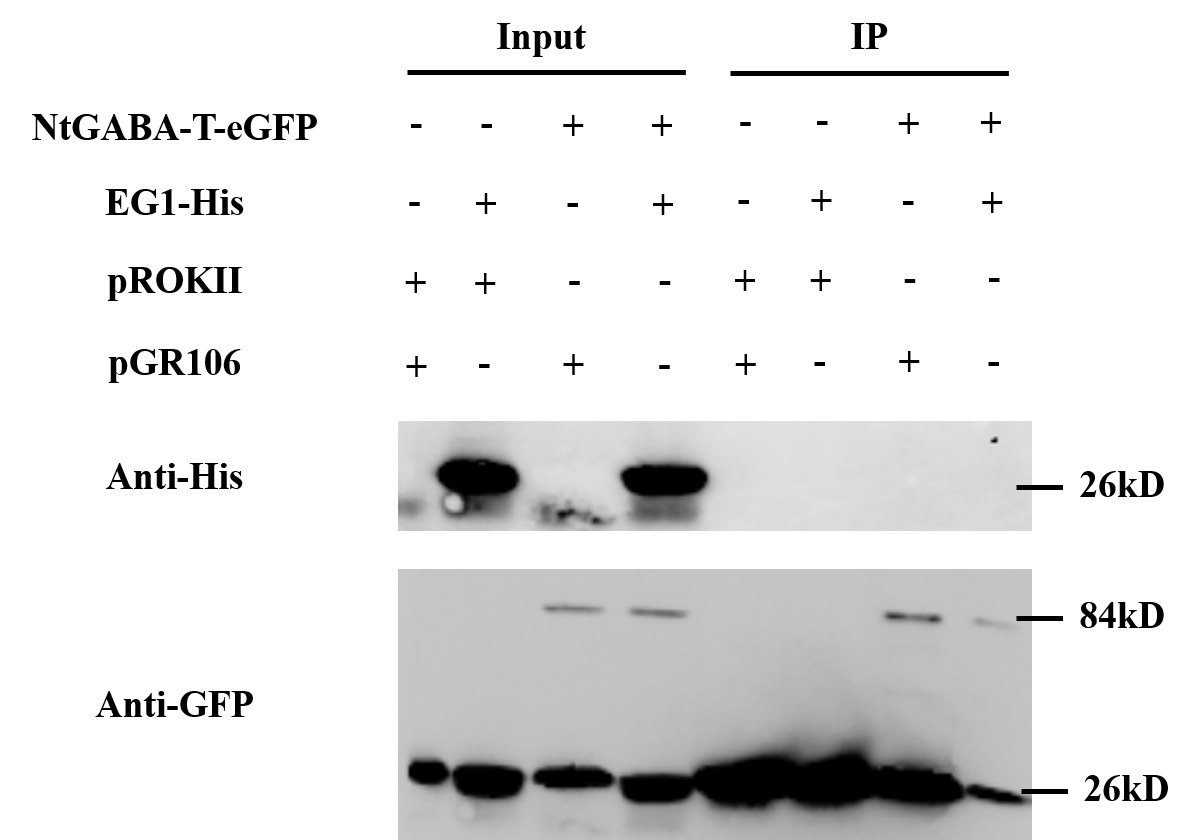


**Supplementary Figure S1.** EG1 does not interact with GABA-T in other species. **(A)** Sequence alignment of GABA-T in *Zea mayz*, *Oryza sativa*, *Arabidopsis thaliana* and *Nicotiana tabacum*. **(B)** Pull-down assay of EG1-His6 and AtGABA-T-eGFP. EG1 stably expressed by *Pichia pastoris* GS115 and purified by Ni-NTA agarose was mixed with AtGABA-T-eGFP transiently expressed by *Nicotiana benthamiana*, then incubated with GFP beads at 4°C overnight, after immunoprecipitation, proteins were detected by western blot using anti-His and anti-GFP antibodies.The experiment was repeated independently three times with similar results. **(C)** Pull-down assay of EG1-His6 and NtGABA-T-eGFP. Methods and results are the same as **(B)**.

**Supplementary Figure S2.**

A


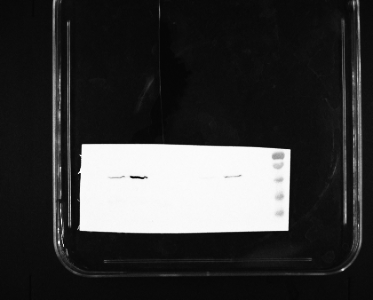


**Anti-HA**

B


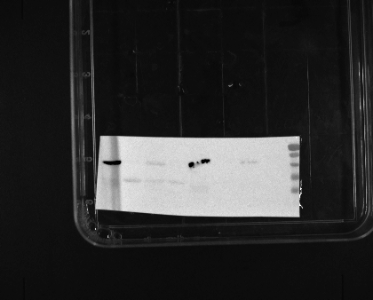


**Anti-GFP**

**Supplementary Figure S2.** Original uncropped blot figures of **Figure 1D. (A)** Anti-HA antibodies were used to detect the HA tag **(B)** Anti-GFP antibodies were used to detect the GFP tag.

**Supplementary Figure S3.**

A


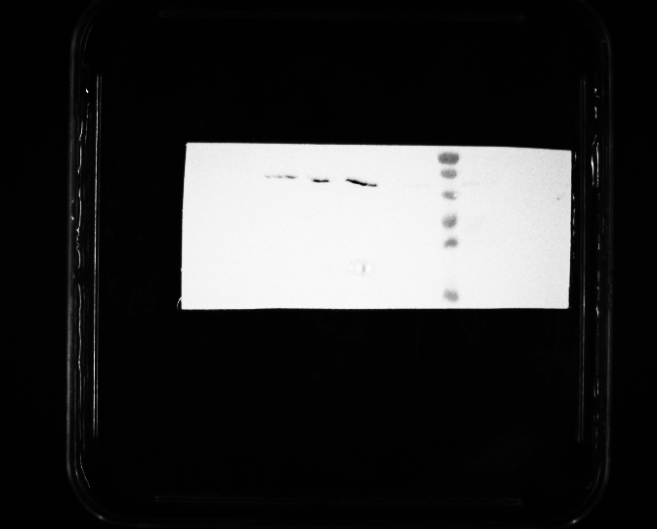


**Anti-HA**

**B**

**
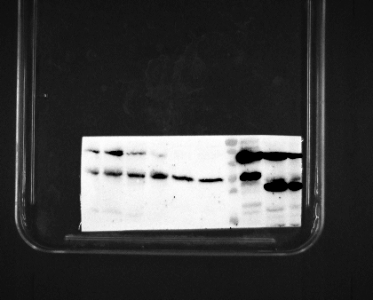
**

**Anti-GFP**

**C**

**
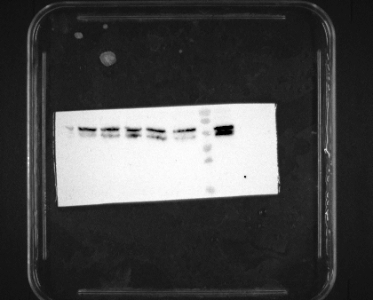
**

**Anti-Actin**

**Supplementary Figure S3.** Original uncropped blot figures of **Figure 3D. (A)** Anti-HA antibodies were used to detect the HA tag **(B)** Anti-GFP antibodies were used to detect the GFP tag. (C) Anti-Actin antibodies were used to detect the plant actin. The red square indicates the destination swimlanes.

**Supplementary Figure S4.**

A

**
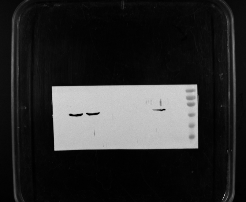
**

**Anti-HA**

B


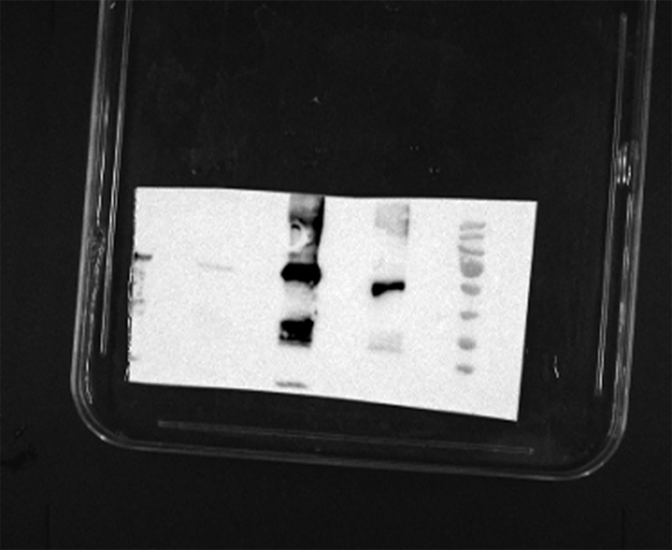


**Anti-GFP**

**Supplementary Figure S4.** Original uncropped blot figures of **Figure 6B. (A)** Anti-HA antibodies were used to detect the HA tag **(B)** Anti-GFP antibodies were used to detect the GFP tag.

**Supplementary Figure S5.**

A


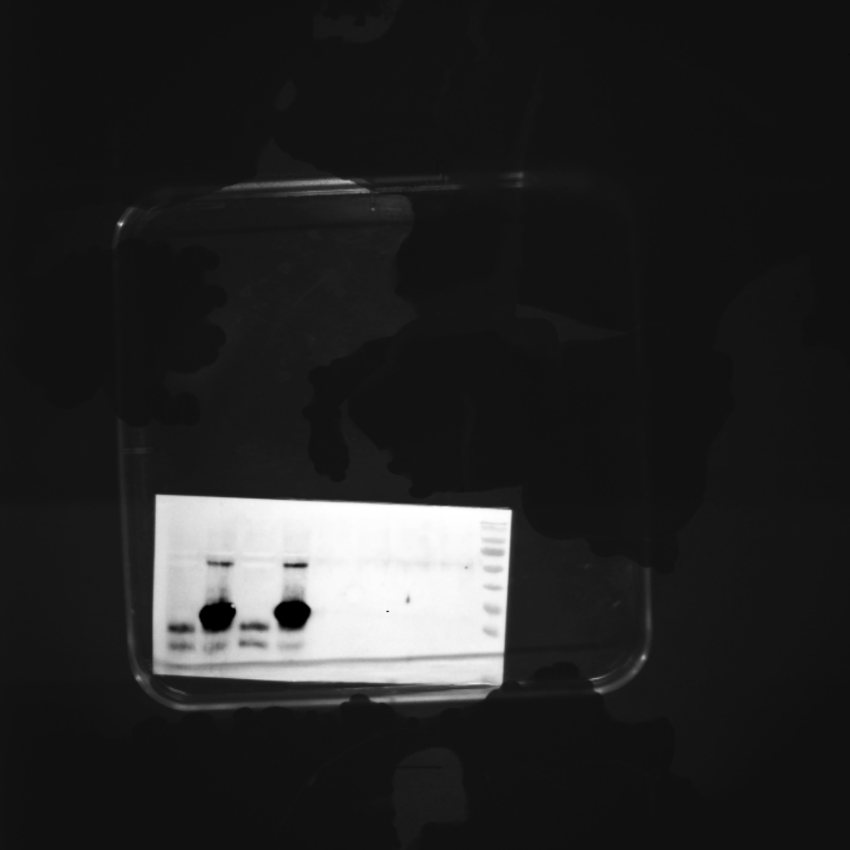


**Anti-His**

B


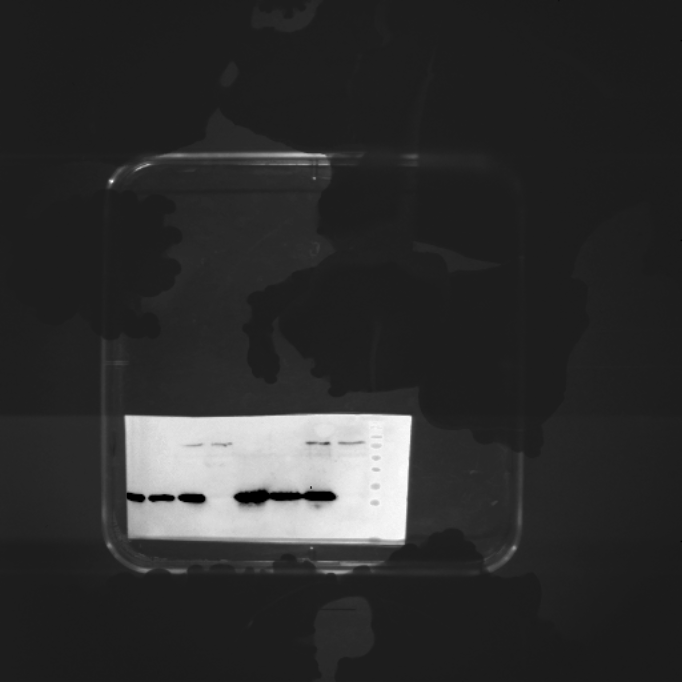


**Anti-GFP**

**Supplementary Figure S5.** Original uncropped blot figures of **Figure S2B. (A)** Anti-His antibodies were used to detect the His tag **(B)** Anti-GFP antibodies were used to detect the GFP tag.

**Supplementary Figure S6.**

A


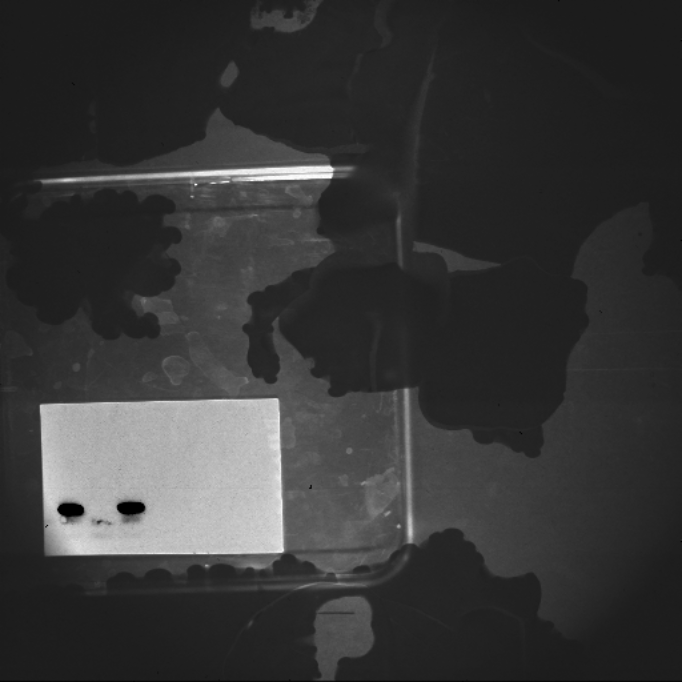


**Anti-His**

B


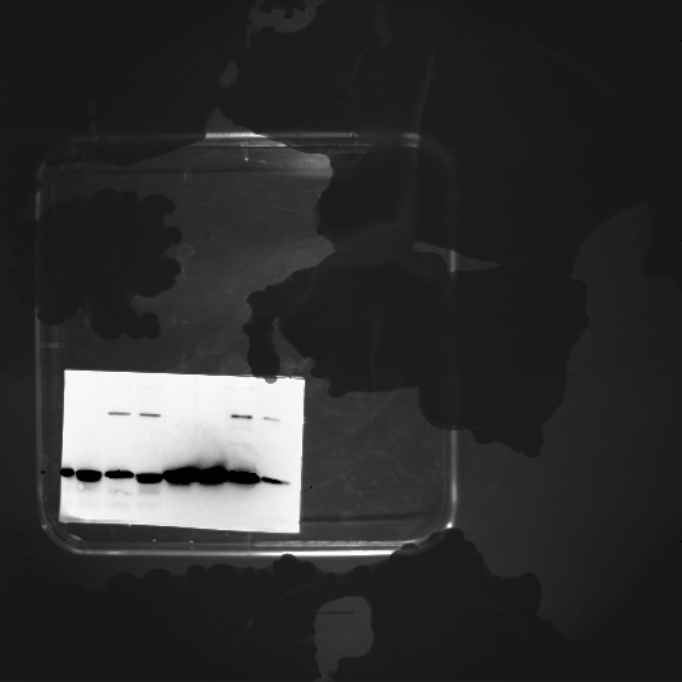


**Anti-GFP**

**Supplementary Figure S6.** Original uncropped blot figures of **Figure S2C. (A)** Anti-His antibodies were used to detect the His tag **(B)** Anti-GFP antibodies were used to detect the GFP tag.
